# Dengue virus replication enhances labile zinc pools by modulation of ZIP8

**DOI:** 10.1111/cmi.13395

**Published:** 2021-10-15

**Authors:** Aleksha Panwar, Jigme Wangchuk, Meenakshi Kar, Rakesh Lodha, Guruprasad R. Medigeshi

**Affiliations:** 1 Clinical and Cellular Virology lab, Infection and Immunology Division, Translational Health Science and Technology Institute, Faridabad, India; 2 Department of Pediatrics, All India Institute of Medical Sciences, New Delhi, India

**Keywords:** dengue virus, replication, viremia, zinc, zinc transporters, ZIP8

## Abstract

Zinc-dependent viral proteins rely on intracellular zinc homeostasis for successful completion of infectious life-cycle. Here, we report that the intracellular labile zinc levels were elevated at early stages of dengue virus (DENV) infection in hepatic cells and this increase in free zinc was abolished in cells infected with UV-inactivated virus or with a DENV replication inhibitor implicating a role for zinc homeostasis in viral RNA replication. This change in free zinc was mediated by zinc transporter, ZIP8, as siRNA-mediated knockdown of ZIP8 resulted in abrogation of increase in free zinc levels leading to significant reduction in DENV titers suggesting a crucial role for ZIP8 in early stages of DENV replication. Furthermore, elevated free zinc levels correlated with high copy numbers of dengue genome in peripheral blood leukocytes obtained from dengue patients compared to healthy controls suggesting a critical role for zinc homeostasis in dengue infection.

**Take Aways:**

- Dengue virus utilises cellular zinc homeostasis during replication of its RNA.
- ZIP8 upregulates free zinc levels during dengue virus replication.
- Enhanced viremia associates with elevated intracellular free zinc in dengue.

## 1 Introduction

Zinc is an essential micronutrient and an important structural constituent of various proteins such as transcription factors, enzymes, growth factors, cytokines and receptors involved in cellular signalling cascade (Fukada, Yamasaki, Nishida, Murakami, & Hirano, 2011). It is the second most abundant transition metal in cellular organism after iron and is an integral part of about 10% of all human proteins (Andreini, Bertini, & Cavallaro, 2011). The total cellular zinc content is in the range of hundreds of micromolar which is mainly constituted by protein-bound form of zinc. The labile (free) zinc pool in cytosol, which is more accessible, is of significant interest as its exchange between the cytosol and subcellular compartments by zinc transporters regulates physiological functions (Frederickson, Koh, & Bush, 2005). This exchange is mediated majorly through two protein families, Zrt, Irt-like proteins (ZIP) and zinc transporters (ZnT). ZIPs are responsible for importing zinc inside the cytosol from extracellular space or from the intracellular compartments while ZnTs transport zinc out of the cytosol into intracellular organelles or into the extracellular space (Kambe, Hashimoto, & Fujimoto, 2014; Maret, 2017). Zinc homeostasis is modulated by external stimuli and redistribution of zinc into tissue spaces as part of acute-phase response may also limit or enhance the availability of zinc which is required for the survival and propagation of the invading pathogen depending on its tissue tropism. Although the cellular level of free zinc is of several orders of magnitude lesser compared to the total zinc or the protein-bound form (Maret, 2013), they represent the zinc pool which is readily available and involved in cellular metabolism.

## 2 Results and Discussion

**Figure 1:**
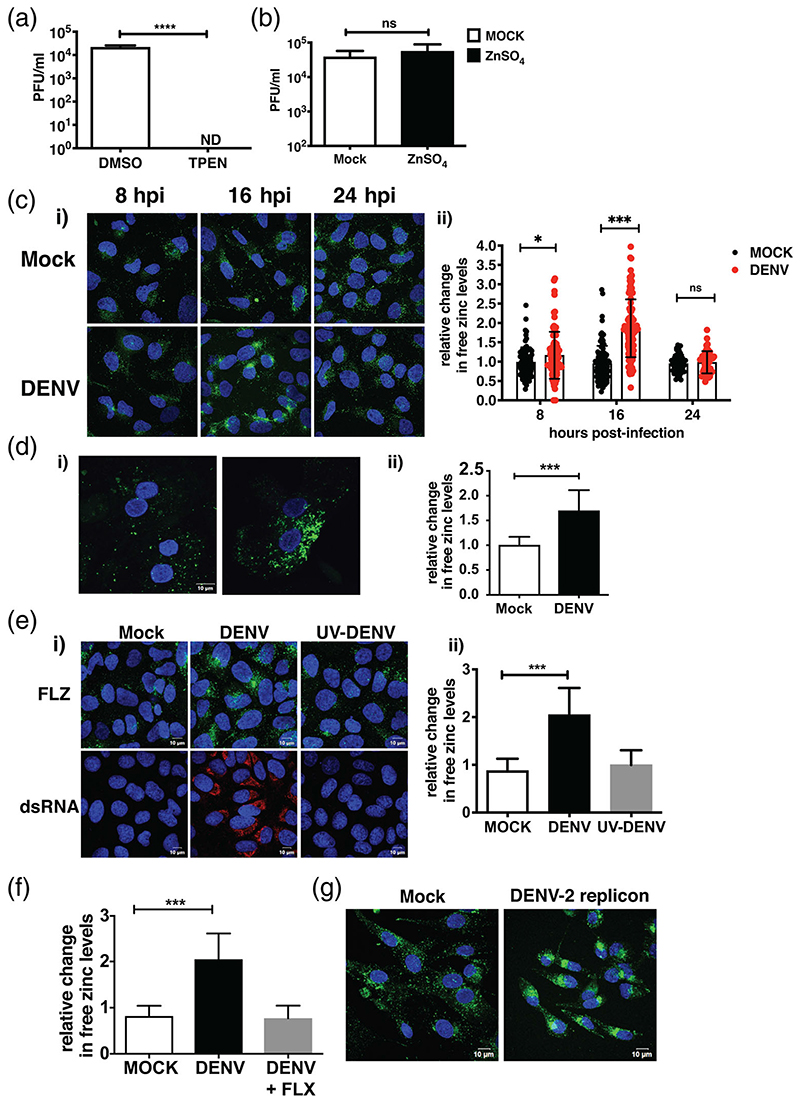
DENV infection leads to increase in cytosolic free zinc levels. Huh-7 cells were infected with DENV at 3 MOI and after viral adsorption, cells were cultured in either 0.5 μM TPEN (a) or 100 μM ZnSO_4_ (b) and viral titers in supernatants was estimated by plaque assay at 24 hpi. (*n* = 9 for both [a] and [b]) (c) Huh-7 cells were infected with DENV and free zinc levels were determined by immunofluorescence assay at indicated time points. (i) Representative images of cells stained with FLZ-3 a.m., (ii) Sum grey intensity of FLZ-3 stain was evaluated using cellSens software. Data represent relative change in sum grey intensity of FLZ-3 a.m. stain in cells (*n* = 95–115) counted from eight different fields. (d) Primary hepatocytes were infected with 5 MOI of DENV and free zinc levels were assessed by immunofluorescence assay at 16 hpi. (i) Representative images of cells stained with FLZ-3 a.m., (ii) Data represent relative change in sum grey intensity of FLZ-3 a.m. stain in cells (*n* = 15–20) counted from seven different fields. (e) Huh-7 cells were either mock-infected or with 3 MOI of DENV or UV-inactivated virus. Cells were stained with FLZ-3 staining at 16 hpi. (i) Representative images (upper panel) and DENV replication was assessed by dsRNA staining using antibody against dsRNA (lower panel), (ii) Data represent relative change in sum grey intensity of FLZ-3 a.m. stain in cells (*n* = 80–100) counted from seven different fields. (f) Huh-7 cells were infected with DENV and fluoxetine hydrochloride (FLX) was added at a concentration of 4 μM after virus adsorption. Data represent fold change in sum grey intensity of FLZ-3 a.m. stain in cells (*n* = 80–100) counted from eight different fields. (g) Free zinc levels were determined in BHK-21 cells stably expressing DENV-2 replicon and mock BHK-21 cells using FLZ-3 a.m. stain. Representative images are shown. All the data presented here are from two or three independent experiments. Data are presented as M ± SD. Scale bar is 10 μM. ns, not significant. **p < .01; ***p < .001

We had shown earlier that zinc chelation by TPEN, a cell-permeable zinc chelator, had a negative effect on dengue infection in epithelial cells (Kar et al., 2019; Khan, Singla, Samal, Lodha, & Medigeshi, 2020). We further confirmed this in Huh-7 cells by infecting cells with DENV at 3 MOI and 1 hr after virus adsoprtion, cells were washed and serum-free medium containing DMSO or 0.5 μM TPEN was added and cultured for 24 hr. Viral titers in the supernatant were measured by plaque assay at 24 hpi. Consistent with our previous findings, we observed that zinc depletion by TPEN led to a significant reduction in DENV titers indicating the importance of zinc in DENV life-cycle (Figure 1a). Similarly, we performed zinc supplementation experiments, where cells were infected as above and serum-free medium containing 100 μM of ZnSO4 was added after virus adsorption and cultured for 24 hr and virus titers were measured by plaque assay at 24 hpi. As observed earlier in epithelial cells (Kar et al., 2019; Khan et al., 2020), supplementing media with excess zinc did not have any effect suggesting that unlike other viruses which are susceptible to inhibition by zinc (Suara & Crowe, 2004; Yuasa et al., 2006; Zhang et al., 1991), DENV infection is not perturbed by excess zinc (Figure 1b). Since DENV replication was sensitive to perturbation in zinc homeostasis and labile zinc pool acts as an indicator of change in zinc homeostasis, we next sought to determine whether DENV infection leads to modulation of zinc homeostasis. Labile zinc levels were visualised by confocal microscopy using zinc fluorophore, fluozin-3 a.m. (FLZ-3 a.m.), in Huh-7 cells infected with DENV at 8 and 16 hpi. We observed a two-fold increase in labile zinc levels in DENV infection at 16 hpi (Figure 1c(i),(ii)). The labile zinc levels at 24 hr were comparable with the mock-infected samples further suggesting that the transient nature of this induction is most likely to meet the demand of viral RNA replication (Figure 1c(i),(ii). The increase in labile zinc levels were found to be MOI-dependent as we observed ~1.5-fold and ~3-fold increase at 0.3 and 3 MOI, respectively, which coincided with exponential phase of virus replication as estimated by qRT-PCR and plaque assay respectively (Figure S1a-c). Further, we verified the above observations in primary human hepatocytes which were infected with DENV at 5 MOI and stained with FLZ-3 a.m. at 16 hpi. Similar to Huh-7 cells, primary hepatocytes showed elevated free zinc levels upon DENV infection (Figure 1d(i),(ii)). We observed ~80–90% infection under these conditions in these primary cells which was similar to what we observe with 3 MOI in Huh-7 cells (Figure S1d). These data suggest that DENV infection modulates zinc homeostasis to meet the demand for zinc in viral replication by inducing elevation in cytosolic free zinc levels in cells of hepatic origin. To verify this, we infected cells with UV-inactivated DENV and measured labile zinc levels at 16 hpi. We found that UV-inactivated virus did not lead to increase in zinc levels as efficiently as an actively replicating virus (Figure 1e(i), (ii)). We next used fluoxetine hydrochloride (FLX), which we have shown earlier to inhibit DENV replication (Medigeshi, Kumar, Dhamija, Agrawal, & Kar, 2016). We observed no change in zinc levels when cells were treated with FLX post-infection (Figure 1f). To further validate this observation, we used BHK-21 cells stably expressing a DENV-2 replicon (Figure S1e) (Boonyasuppayakorn, Reichert, Manzano, Nagarajan, & Padmanabhan, 2014). Mock BHK-21 cells and cells stably expressing DENV-2 replicon were stained with FLZ-3 a.m. to visualise labile zinc levels. (Figure 1g). Similar to virus infection in Huh-7 cells, we observed a significant increase in labile zinc levels in cells expressing the DENV replicon suggesting that active replication of DENV leads to increase in labile zinc pools in the cytosol.

**Figure 2:**
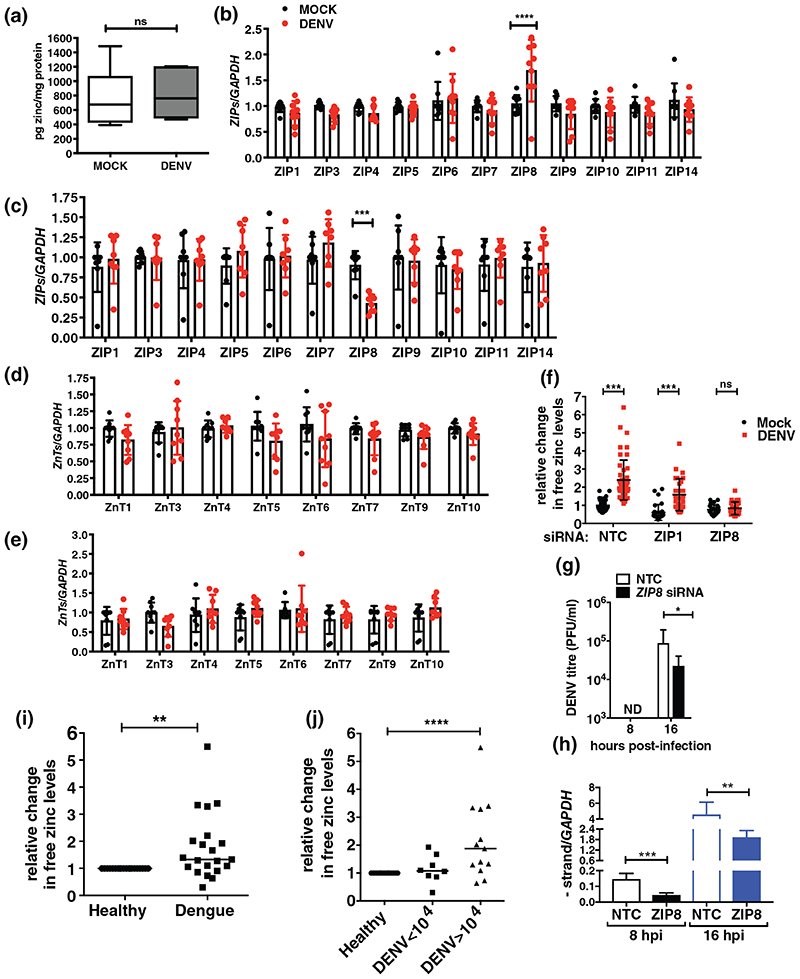
*ZIP8* mediates the increase in intracellular free zinc levels in DENV infection. (a) Huh-7 cells were infected with 3 MOI of DENV and the total content of the free zinc was determined at 16 hpi by ICP-MS (*n* = 6). (b) Huh-7 cells were infected with DENV and cells were collected for RNA isolation. Expression of zinc transporters were assessed by qRT-PCR. (b, c) Graph shows the expression levels of *ZIPs* at 8 and 16 hpi, (d,e) Graph shows the expression levels of *ZnTs* at 8 and 16 hpi. *GAPDH* mRNA levels were used for normalisation (*n* = 9). (f) Huh-7 cells were treated with siRNAs specific to *ZIP-1, ZIP-8* and a non-targeting control (NTC) siRNA. Cells were infected with DENV at 48 hr posttransfection and FLZ-3 a.m. staining was performed at 16 hpi. Graph indicates fold change in sum grey intensity of FLZ-3 a.m. of cells (*n* = 30–35) counted from six different fields. (g) DENV titers in the supernatants at indicated time-points post-infection in *ZIP8* knockdown conditions (*n* = 7). (h) DENV negative strand RNA was estimated at indicated time points by qRT-PCR. *GAPDH* mRNA levels were used for normalisation (*n* = 7). (i) Peripheral blood samples from healthy volunteers (*n* = 20) and dengue patients (*n* = 21) were processed as described in the experimental procedures and stained using FLZ-3 a.m. by flow cytometry. (j) Graphs for FLZ-3 a.m. levels were plotted based on viral load. Samples were categorised as low viral load (<10^4^ DENV RNA copies/ml) and high viral load (>10^4^ DENV RNA copies/ml). Fold change in MFI of FLZ-3 a.m. staining in dengue samples were normalised to healthy control samples. Data in (a)–(h) are presented as *M* ± *SD* and median values are shown in (i) and (j). ns, not significant. *p < .05, **p < .01, ***p < .001, ****p < .0001

**Figure 3:**
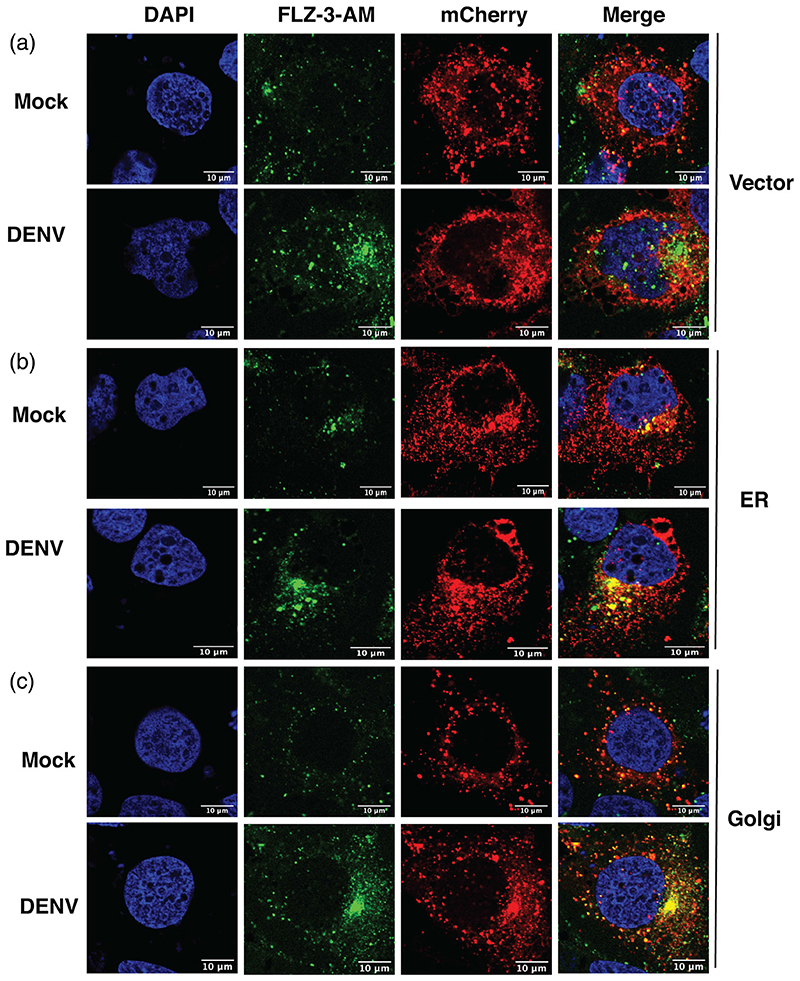
Co-localization of labile zinc pool with endoplasmic reticulum and Golgi. (a) Huh-7 cells were transfected with plasmids expressing mCherry-tagged ER and Golgi-resident marker proteins. After 24 hr post transfection, cells were infected with DENV at 3 MOI and fluozin-3 a.m. staining was performed at 16 hpi. Confocal images were captured using FV3000 microscope. (DAPI: blue; FLZ-3 a.m.: green; organelles: red). (a) Vector, (b) Endoplasmic reticulum (ER), (c) Golgi. Data are from three independent experiments. Single Z-slice is shown. Scale bar is 10 μM

We next sought to identify the mechanism of increase in labile zinc levels in dengue infection. Free zinc levels in the cytoplasm may increase due to enhanced cellular uptake of zinc or due to redistribution of zinc from intracellular organelles to cytosol or by the release of zinc from metallothioneins which act as zinc stores (Kimura & Kambe, 2016). Huh-7 cells were infected with DENV and at 16 hpi, total cell extracts were subjected to inductively coupled-plasma mass spectrometry (ICP-MS) for the detection of total zinc (Zn) content. The amount of zinc ions detected by ICP-MS in parts per billion (ppb) was normalised to the total protein content of the cells to account for any difference in cell numbers. We did not observe any change in the total zinc content in DENV infection conditions (Figure 2a). Flavivirus replication utilises cytosolic membranous compartments derived from the ER and Golgi (Mackenzie, 2005). Therefore, we were interested to examine whether this free zinc pool is localised to flavivirus replication compartments. However, localization of free zinc with viral proteins and viral replication compartment was not possible as permeabilization of cells led to loss of FLZ-3 a.m. signal. Therefore, we transfected plasmid constructs coding for mCherry-tagged ER or Golgi-resident proteins in Huh-7 cells followed by infection with DENV after 24 hr and visualised free zinc levels by confocal microscopy at 16 hpi. We consistently observed higher levels of free zinc in infection conditions which concentrated in the perinuclear region as observed earlier. This perinuclear free zinc pool colocalized with mcherry-ER and mCherry-Golgi in DENV infection conditions suggesting indirectly that the free zinc pool may be closely associated with DENV replication compartments derived from the ER and Golgi (Figure 3).

We speculated that the increase in cytosolic zinc induced by dengue virus infection could be regulated by zinc transporters. To test this, we determined the mRNA expression levels of each members of *ZIP* and *ZNT* families upon DENV infection at 8 and 16 hpi (Figure 2b–e). *ZIP2*, *ZIP12* and *ZIP13* and *ZNT2* and *ZNT8* mRNA levels were below the limit of detection of the assay. Among all the *ZIPs* analysed, *ZIP8* expression was significantly upregulated in DENV infection at 8 hr and showed downregulation at 16 hr. None of the other *ZIP*s or *ZNT*s analysed in the study showed any modulation under these conditions. Therefore, ZIP8 upregulation at early stages of infection may contribute to increase in free zinc levels in DENV infection. We hypothesized that downregulation in *ZIP8* expression at 16 hr could be a cellular response to negate further increase in cytosolic zinc levels which could be cytotoxic. To ascertain the importance of ZIP8 in dengue infection-induced increase in labile zinc levels, we performed siRNA-mediated knockdown of *ZIP8* in Huh-7 cells. *ZIP1*, which is a ubiquitous ZIP, was selected as a specificity control (Figure S2a). Huh-7 cells were infected with DENV at 48 hr post-transfection with siRNAs and processed for FLZ-3 a.m. staining at 16 hpi. We observed a significant increase in free zinc levels in non-targeting control (NTC) and *ZIP1* knockdown (KD) conditions. However, *ZIP8* knock-down abrogated the increase in labile zinc levels observed in DENV infection further underscoring the role of *ZIP8* in this event (Figures 2f & S2b). Knock-down of *ZIP8* levels led to ~50% reduction in DENV titers (Figure 2g). DENV non-structural protein-5 is a metalloenzyme binding zinc (El Sahili & Lescar, 2017; Yap et al., 2007) and DENV RNA replication proceeds via negative strand intermediates. Therefore, measuring negative strand of DENV RNA provides a more accurate estimate of active DENV replication. We measured the negative strand RNA levels in control or *ZIP8* siRNA-treated and DENV-infected cells by qRT-PCR. We found about threefold reduction in the amount of negative strand intermediates in *ZIP8* knock-down cells as compared to NTC at both 8 and 16 hpi (Figure 2h). These results clearly indicate that ZIP8 is involved in the regulation of free zinc levels during DENV replication. Zinc is an acute-phase reactant which undergoes tissue redistribution because of increased uptake by liver. In the case of DENV, the primary cell types in blood that are susceptible to infection are the dendritic cells, monocytes and B cells (King et al., 1999; Schmid, Diamond, & Harris, 2014; Valero et al., 2014). Based on our in vitro data, we next probed whether blood cells from dengue patients show an increase in labile zinc levels. We recruited 21 paediatric dengue patients who were in the viremic phase (within 7 days of fever) into the study to collect blood samples and analyse for free zinc levels in peripheral blood cells by flow cytometry. The clinical features of these patients have been reported recently as part of another study (Khan et al., 2021). As per WHO classification guidelines, 8 patients had mild dengue, 7 had dengue with warning signs and 6 were with severe dengue. Total RNA was isolated from whole blood and processed for estimation of viral RNA copy numbers by qRT-PCR as described previously (Kar et al., 2017; Khan et al., 2021; Singla et al., 2016). We also included healthy volunteers for relative comparison of labile zinc levels by flow cytometry. We measured the labile zinc levels in total peripheral blood lymphocytes (PBLs) by flow cytometry. Fold change in mean fluorescent intensity (MFI) of dengue samples was normalised to healthy control samples. The labile zinc levels were higher in PBLs from dengue samples relative to healthy controls (Figure 2i). Further, we categorised the dengue samples based on the copy numbers of viral genome per ml of blood as (a) samples having less than 10^4^ genome copy numbers/ml of blood (*n* = 8) and (b) samples having more than 10^4^ genome copy numbers/ml of blood (*n* = 13). We observed a significant increase in labile zinc levels in samples with high DENV viral genome copy numbers (Figure 2j). Collectively, these findings indicate that elevated free zinc levels are associated with DENV replication in Huh-7 cells and with dengue viremia in patients establishing a link between zinc homeostasis and DENV infection.

Our results indicate a potential role for zinc in DENV life-cycle particularly during early stages of replication. The DENV RNA polymerase, which is a zinc metalloenzyme, may sequester free zinc to viral replication compartments to cater to the requirement of zinc in replication of viral RNA. Therefore, any interventions that prevent supply of zinc to replication sites would perturb DENV replication as observed by knock-down of *ZIP8* which led to reduction in negative strand intermediates generated during DENV RNA replication. We speculate that redistribution of free zinc to DENV replication sites may act as a trigger for infected cells to compensate for this loss of cytosolic free zinc by recruitment of zinc from cellular zinc stores by ZIP8. ZIP8 is localised on the plasma membrane, lysosome and mitochondrial membrane (Aydemir, Liuzzi, McClellan, & Cousins, 2009; Besecker et al., 2008) and is upregulated as a downstream effector of NF-κB signalling (Liu et al., 2013). Interestingly, *ZIP8* induction leads to increase in intracellular zinc levels which acts as a negative feedback loop to inhibit NF-κB pathway and stop the inflammatory process. We need to further explore whether NF-κB pathway has a role in downregulation of *ZIP8* at later stages of infection as observed in our study. Another study has shown that inhibition of NF-κB by zinc was mediated by cGMP accumulation and activation of protein kinase A (PKA) (von Bulow et al., 2007). Interestingly, we have shown previously that treating cells with an inhibitor of PKA led to inhibition of dengue virus replication (Kumar, Agrawal, Khan, Nakayama, & Medigeshi, 2016). Therefore, these data suggest that increase in labile zinc levels may not only aid DENV replication, but may also suppress antiviral responses to facilitate viral RNA replication. We need to further determine the role of NF-κB-ZIP8 axis in dengue virus replication to further understand the signalling pathways that are regulated by labile zinc pools. During acute-phase response, zinc uptake is enhanced in the liver which may create an ideal niche for hepatotropic viruses such as dengue virus that depend on zinc for productive replication. Recent reports have also shown that zinc inhibits interferon-*λ*3 signalling in Huh-7 and other cell types and high zinc correlated with lower inflammation and higher viral load in patients infected with hepatitis C virus (Read et al., 2017). We observed a similar association between dengue viremia and intracellular zinc levels in peripheral blood leukocytes suggesting a link between zinc homeostasis and dengue disease outcomes. Whether this pathway plays a prominent role in liver pathogenesis observed with other viruses warrants further investigation and may provide useful insights into the role of zinc homeostasis in viral infections.

## 3 Experimental Procedures

List of reagents used in the study is provided in Table S1.

### 3.1 Screening and enrolment of dengue patients

The study was approved by the Institutional Ethics committees of both the participating institutes (IEC/NP-352/08.10.2014 and THS 1.8.1/[32] dt. fifth April 2015). All patients in this study were enrolled between September 2016 and January 2019 at the Department of Paediatrics, All India Institute of Medical Sciences (AIIMS), New Delhi. Screening and enrolment of patients were exactly as described previously (Kar et al., 2017; Khan et al., 2021; Singla et al., 2016). Written informed consent for the study was taken from parents/guardians to collect blood samples at the time of admission. Children aged between 4-14 years with symptoms suggestive of dengue were screened using a “Dengue Day 1 Test” which is a rapid solid phase immunochromatographic test for the qualitative detection of dengue NS1 Antigen (J. Mitra and Co. Pvt. Ltd, New Delhi). Patients testing positive for either NS1 or IgM were informed and requested for consent for the study. Informed consent was also obtained from young adult volunteers to collect blood samples for healthy controls (age 18–30 years; 9 Male, 7 Female). Complete blood counts were performed on automated COULTER COUNTER analyser (Beckman Coulter).

### 3.2 Cells and viruses

Huh-7 were propagated as described previously (Kar et al., 2019; Medigeshi et al., 2016). Huh-7 cells were grown in Dulbecco’s Modified Eagle’s medium (DMEM) containing 1 g/L glucose supplemented with 10% fetal bovine serum (FBS) and 100 units penicillin, 100 μg streptomycin, 2 mM L-glutamine (1X PSG-Gibco). Primary hepatocytes (ThermoFisher Scientific) were grown in William’s E medium with thawing and plating supplements (ThermoFisher Scientific). BHK-21 cells were grown in Minimum Essential Medium (MEM-Gibco) supplemented with 10% FBS and 1X PSG. BHK-21 cells stably expressing DENV-2 luciferase replicon (Boonyasuppayakorn et al., 2014) and control cells were grown in DMEM containing 4.5 g/L glucose, supplemented with 10% FBS, 1X PSG and 1X Non-Essential Amino Acids (NEAA-Gibco). Huh-7 cells were infected with 3 MOI of DENV-2 (New Guinea C strain) in respective growth media containing 2% FBS for 1 hr. After virus adsorption, cells were washed twice with 1X PBS (Phosphate buffered saline) and growth medium containing 10% FBS was added to the cells. Primary hepatocytes were grown on collagen-coated surface and infected with DENV-2 at 5 MOI. After virus adsorption, cells were washed with 1X PBS and 5% serum containing medium was added. Virus infection was confirmed by plaque assay in BHK-21 cells as described previously (Agrawal, Schu, & Medigeshi, 2013) or by intracellular staining for dsRNA or dengue envelope protein by immunofluorescence.

### 3.3 Detection of free zinc levels by confocal microscopy

FluoZin-3 a.m. (FLZ-3 a.m.) (Invitrogen) was used to determine the intracellular free zinc levels. Medium was removed and cells were washed twice with 1X PBS followed by incubation with FLZ-3 a.m. at a concentration of 10 μM. Cells were incubated for 20 min at 37°C. After incubation, cells were washed three times with 1X PBS and stained with 4’,6-diamidino-2-phenylindole (DAPI) at a dilution of 1:10,000 in PBS for 5 min. Cells were fixed using 3% paraformaldehyde (PFA) for 10 mins followed by washing with 1X PBS three times and mounted using Prolong Gold antifade reagent (Invitrogen). Images were captured using FV3000 confocal microscope (Olympus). Fluorescence images with FLZ-3 a.m. were captured using an excitation wavelength of 488 nm. The parameters for detection and capturing images were digitally controlled to keep same settings throughout the experiments. For quantitative analysis, sum grey intensity per cell was calculated using CellSens software (Olympus) and plotted as relative change.

### 3.4 Treatment of cells

To study the effect of *N*, *N*, *N*′, *N*′-tetrakis(2-pyridinylmethyl)-1,- 2-ethanediamine (TPEN) (Sigma) on virus infection, 0.5 μM TPEN was added in serum-free media after 1 hr of viral adsorption. At 24 hpi, supernatant was collected for estimating virus titre by plaque assay. For zinc supplementation experiment, 100 μM of ZnSO_4_ was added in serum-free media after viral adsorption and viral titers were measured at 24 hpi. To study the effect of fluoxetine hydrochloride on DENV-induced free zinc levels, Huh-7 cells were infected with DENV and fluoxetine hydrochloride was added after viral adsorption at a concentration of 4 μM in 2% DMEM. Cells were incubated for 16 hpi and processed for FLZ-3 a.m. staining. Coverslips were mounted on glass slides, sealed and images were captured at 100X using FV3000 confocal microscope (Olympus).

### 3.5 Transient transfections

Huh-7 cells were seeded at 60,000 cells per well in a 24-well plate on glass coverslips. 24 hr later, cells were washed with 1X PBS, and transfected with 800 ng of plasmids expressing mCherry-tagged endoplasmic reticulum (Addgene-55,041) and Golgi (Addgene-55,052) markers in serum-free media using Lipofectamine2000 (Invitrogen) as per the manufacturers’ instructions. After 6 hr incubation period, the transfection mixtures were removed and replaced with fresh growth medium. After 24 hr post transfection, cells were infected with DENV at 3 MOI. At 16 hpi, cells were stained with FLZ-3 a.m. and processed for confocal microscopy as described in the previous sections.

### 3.6 Viral dsRNA staining

At 16 hpi, cells were washed with cold PBS and fixed in ice-cold methanol at −20°C for ≥20 min. Cells were washed twice with PBS followed by incubation in 0.2% BSA in IMF buffer (20 mM HEPES pH 7.5, 0.1% Triton X-100, 150 mM NaCl, 5 mM EDTA, 0.02% sodium azide) for 1 hr at room temperature (RT). Cells were then incubated with J2 antibody (English and Scientific) at a dilution of 1:1,000 in the blocking buffer for 1 hr at RT. Cells were washed thrice with IMF buffer followed by incubation with Alexa Fluor 568-tagged secondary antibody (Invitrogen) at a dilution of 1:500 in blocking buffer for 30 minutes at RT in dark. Cells were washed thrice with IMF buffer and stained with DAPI at a dilution of 1:10,000 for 5 min. Cells were washed with 1X PBS, mounted using Prolong Gold antifade reagent and images were captured using FV3000 confocal microscope.

### 3.7 Western blot

BHK-21 cells expressing DENV-2 replicons were grown in 24 wellplate. Cell lysates were prepared at 24 hr post seeding by washing cells twice with cold PBS on ice and cells were lysed by scraping cells into 100 μl of RIPA buffer (50 mM Tris-HCl pH 8.0, 150 mM NaCl, 1% IGEPAL CA-630, 0.5% sodium deoxycholate, 0.1% SDS with protease inhibitor mix [Roche] and 1 mM PMSF). Lysates were incubated on ice for 10 min and centrifuged at 13,000g for 15 min at 4°C. Supernatants were boiled in 1X Laemmli buffer and resolved on 10% SDS-PAGE. Gels were transferred onto PVDF (GE Healthcare) membranes for 2 hr and analysed by probing the blot with the DENV non-structural protein 3 (NS3) antibody and β-actin was used as housekeeping control. Primary antibodies incubation was followed by secondary antibodies tagged with Alexa fluor 488 and 633 (Invitrogen). Signals were detected using a gel documentation system (Azure biosystems C400).

### 3.8 Ultraviolet (UV)-inactivation of DENV

DENV inactivation was carried out using a UV crosslinker (UVP-CL1000S). DENV stock was diluted in a ratio of 1:100 in 1 ml of MEM media and placed in a 35 mm diameter petri dish. DENV stock was treated with a dose of UV light at 9999 × 10^2^ μJ/cm^2^. The supernatant was exposed three times for 10 min with an interval of 3 min. After the inactivation process, virus stock was aliquoted and stored at −80°C, later titered and used for the experiment. UV-inactivated virus showed no replication as observed by viral dsRNA staining.

### 3.9 Measurement of zinc content by ICPMS

Huh-7 cells were seeded in 12 well plate and infected with DENV. At 16 hpi, cells were washed twice with 1X PBS and collected in 1 ml of 0.1% SDS prepared in ultrapure water (Sigma). The lysate was filtered using 0.45 μm pore polytetrafluoroethylene (Teflon) membrane filter (Cole Parmer—15945-41). Multielement standards (Merck) were used from 500 ppb to 1.95 ppb using the two-fold dilution method in deionised water. Acquisition was carried out using the X series two inductively coupled plasma mass spectrometer (ICP-MS; Thermo Fisher Scientific). Protein estimation was carried out using a bicinchoninic acid (BCA) kit (Pierce). Data were normalised to total protein content to account for any difference in cell numbers and represented as pg zinc per mg of protein.

### 3.10 FluoZin-3-The AM staining in dengue patients

Whole blood was collected in Na-Citrate tubes (around 4 ml) and centrifuged at 50g for 15 min with no brakes to separate platelet-rich plasma (PRP). PRP was removed and remaining blood was subjected to RBC lysis using RBC lysis buffer (155 mM NH_4_Cl, 12 mM NaHCO_3_, 0.1 mM EDTA) added at a ratio of 1:10 (1 ml blood and 10 ml RBC lysis buffer). Cells were incubated for 15 min at RT in dark followed by centrifugation at 200g for 10 min. Cell pellet was washed twice with PBS by centrifugation at 200g for 10 min. Cell viability and counts were estimated by trypan blue staining. 1 million cells were stained using FLZ-3 a.m. in DMEM without phenol red supplemented with 2 mM L-glutamine (staining medium) in a final volume of 100 μl. Cells were incubated at 37°C in a CO_2_ incubator for 30 min and washed with DMEM without phenol red at 500g for 5 min. Fixable viability stain eFluor780 was used at 1:250 dilution. Cells were washed with FACS buffer and fixed using IC fixation buffer (eBiosciences). Cells were acquired in FACS Canto II (Becton Dickinson). Peripheral blood lymphocytes (PBLs) were gated using SSC-A vs FSC-A gate which were further gated based on granulocyte marker CD66b. CD66b-negative population (lymphocytes) was gated for live cells using fixable viability stain eFluor780. The amounts of labile zinc present in live cells was estimated in FITC channel and were presented as the mean fluorescence intensities of FLZ-3-AM.

### 3.11 siRNA knockdown

Smartpool siRNAs targeting the human *ZIP1* and *ZIP8* genes or nontargeting controls (NTC) were purchased from Dharmacon. Transfections were carried out as described previously (Kumar et al., 2016). All transfections were performed as per manufacturer’s instructions. Briefly, 10 nM concentrations of NTC, ZIP1 and *ZIP8* siRNAs were mixed with Opti-MEM (Life Technologies) and 1 μl of Lipofectamine RNAiMax to a total volume of 100 μl in a 24-well plate. Cells were trypsinized and volume made up so as to contain 30,000 cells in 400 μl antibiotic-free medium. After 20 min incubation of the transfection complex, cell suspension was added into each well. Knockdown efficiency was determined by qRT-PCR at 48 hr post transfection.

### 3.12 Quantitative real time PCR (qRT-PCR)

Huh-7 cells were infected with DENV and at indicated time points, cells were collected in TRIzoL reagent (Takara) and RNA was isolated using manufacturer’s instructions. cDNA synthesis was performed using PrimeScript RT reagent kit with gDNA eraser (Takara). 100 ng of cDNA was used to determine genes expression using DyNAmo flash SYBR green quantitative PCR reagent (Thermo Scientific). Reaction conditions used were as follows: (95°C-7 min; 95°C-10 s followed by 60°C for 30 s). GAPDH primer was used as housekeeping control. For DENV RNA detection by reverse-transcription polymerase chain reaction (RT-PCR), total RNA was extracted from cells at the indicated time points using RNAiso Plus (TaKaRa), and 200 ng of RNA was used in multiplex TaqMan one-step RT-PCR with DENV primers, DENV probe and human GAPDH primer-probe mix (Applied Biosystems). At indicated time points, supernatant was collected for estimating viral titers by plaque assay and cells were harvested for positive and negative strand detection PCR as described previously (Kar et al., 2017). Expression levels of GAPDH was used to calculate fold change and normalisation. Data were analysed using the ΔΔ C_T_ method, where C_T_ is threshold cycle.

### 3.13 Data analysis

Data were analysed and graphs were prepared using Prism 7 (Version 7.0e) software (GraphPad Software Inc.). All the graphs represent results from two or more independent experiments; values are presented as *M* ± *SD*. Statistical significance was estimated by Mann-Whitney test. The data were corrected for multiple comparisons using Bonferroni-Dunn method wherever applicable.

## Supplementary Material

**Supporting Information**

Additional supporting information may be found in the online version of the article at the publisher’s website.

Figure S1

Figure S2

Table S1

## Data Availability

All the data presented in the manuscript is available as part of this manuscript and as supplementary information.
