# Supplementary figures and images for: Dengue virus replication enhances labile zinc pools by modulation of ZIP8
Source: Cell Microbiol. 2021 Oct 15;23(12):e13395. doi: 10.1111/cmi.13395 (PMC7612096; doi:10.1111/cmi.13395)

Supplementary Figures

Figure S1

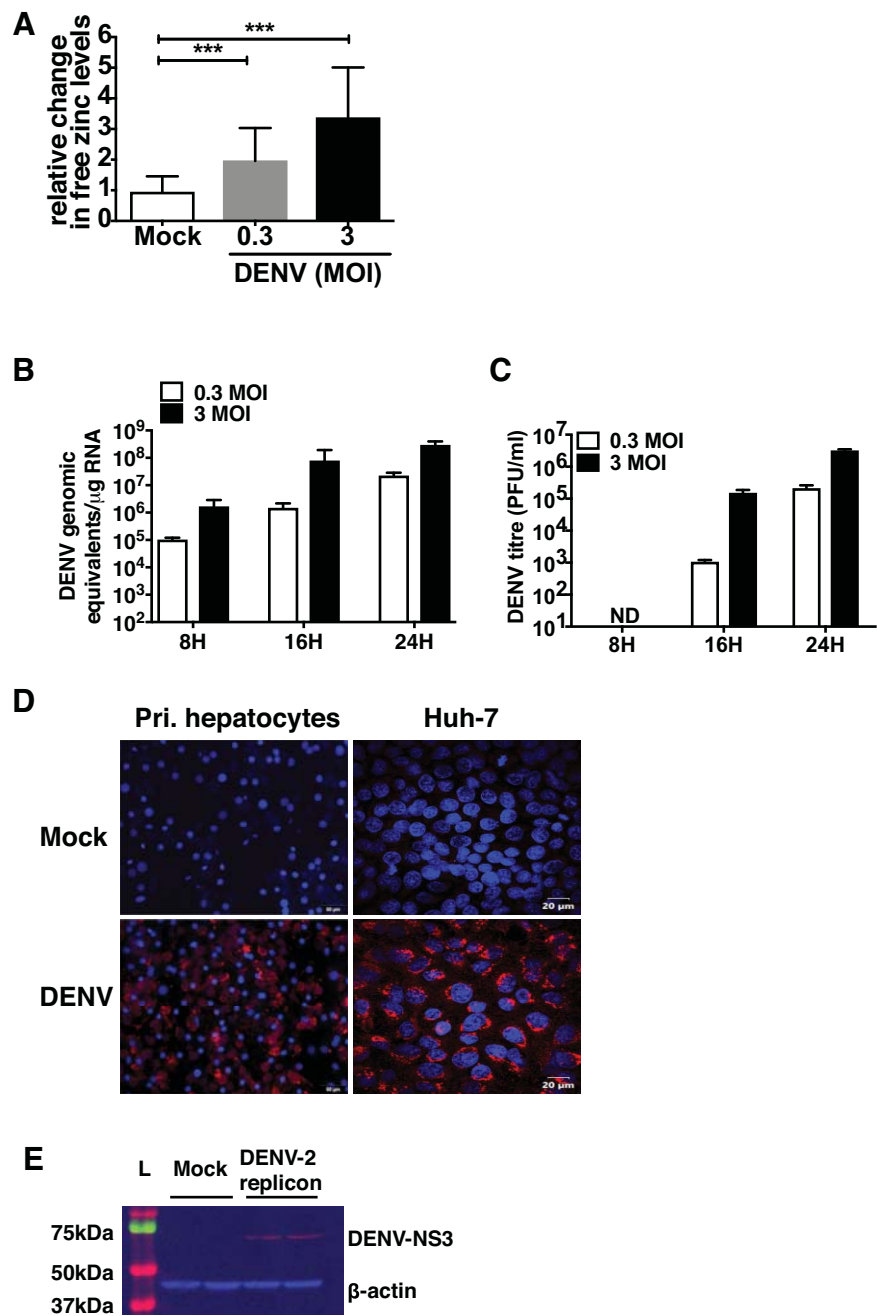

Supplement: Supplementary file 1 — Figure S1. MOI‐dependent increase in labile zinc levels. (a) Huh‐7 cells were infected with DENV at 0.3 and 3 MOI and free zinc levels were visualised using FLZ‐3 a.m. at 16 hpi. Graph indicates fold change in sum grey intensity of FLZ‐3 a.m. of cells (n = 35–50) counted from six different fields. (b) Viral RNA copy numbers were assessed by qRT‐PCR at indicated time points. (c) DENV titers were measured in the supernatants by plaque assay. (d) Primary hepatocytes or Huh‐7 cells were infected with DENV at 5 and 3 MOI respectively. Cells were fixed in methanol at 24 hpi and DENV percent infection was determined using DENV‐envelope antibody by immunofluorescence assay. Representative images are shown. (e) Western blot indicating expression of DENV‐NS3 in mock or cells expressing DENV‐2 replicon. Data are from at least two independent experiments. Data are presented as M ± SD. Scale bar is 50 μM. ***p < .001 [file CMI-23-e13395-s003.pdf]

Figure S2

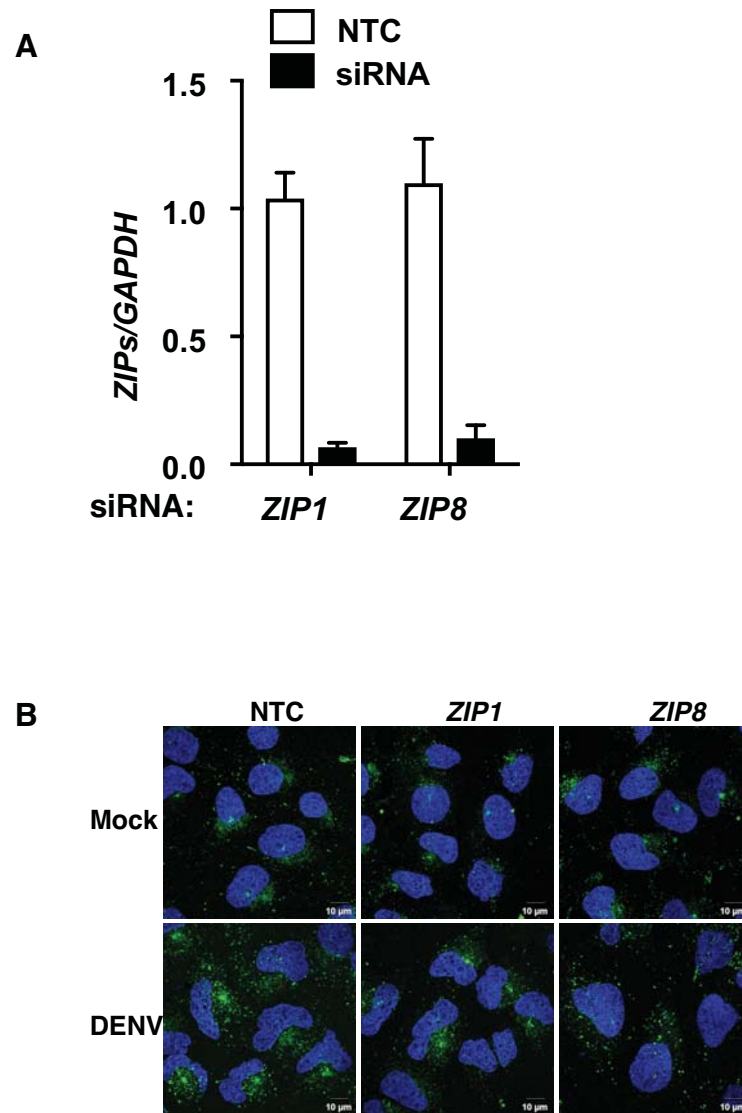

Supplement: Supplementary file 2 — Figure S2. ZIP8 knockdown abrogates free zinc increase in DENV infection. Huh‐7 cells were treated with siRNA specific to ZIP‐1, ZIP‐8 and scrambled siRNA as a control (labelled as NTC (nontargeted control). (a) Graph indicates the knockdown efficiency determined at the mRNA level by qRT‐PCR at 48 hr post transfection. (b) Huh‐7 cells were infected with DENV at 48 hr post transfection. FLZ‐3 a.m. staining was performed at 16 hpi. Representative images are shown. Scale bar is 10 μM [file CMI-23-e13395-s002.pdf]
