# Supplementary material for: Dengue virus replication enhances labile zinc pools by modulation of ZIP8
Source: Cell Microbiol. 2021 Oct 15;23(12):e13395. doi: 10.1111/cmi.13395 (PMC7612096; doi:10.1111/cmi.13395)
Supplement: Supplementary file 3 — Table S1. List of reagents used in the study [file CMI-23-e13395-s001.docx]

Supplementary Table S1: List of reagents used in the study

| REAGENT or RESOURCE SOURCE IDENTIFIER |
| --- |
| **Antibodies** |
| Anti dsRNA monoclonal English & Scientific Cat. No. J2 |
| DENV non-structural protein 3 Kind gift from Raj Bhatnagar (Kakumani et al., 2015) |
| DENV-Envelope antibody (Hybridoma) ATCC Cat.No.HB-112 |
| β-actin Sigma-Aldrich Cat. No. A2228 |
| Goat anti-mouse AF488 Thermo Fisher Cat. No. A21202 |
| Goat anti-mouse AF568 Thermo Fisher Cat. No. A11019 |
| Goat anti-rabbit AF633 Thermo Fisher Cat. No. A10523 |
| **Virus strains** |
| DENV-2 New Guinea C Strain Kind gift from Dr. Anmol Chandele |
| **Biological Samples** |
| Blood samples from dengue patients All India Institute of Medical Sciences (AIIMS),  New Delhi, |
| **Chemicals, peptides and recombinant proteins** |
| *N, N, N*′, *N*′-tetrakis(2-pyridinylmethyl) Sigma-Aldrich Cat. No. P4413  -1,2-ethanediamine (TPEN) |
| Zinc sulfate (ZnSO_4_) Sigma-Aldrich Cat. No. Z0251 |
| Human SLC39A1(ZIP1) siRNA Dharmacon Cat. No. 27173 |
| Human SLC39A8(ZIP8) siRNA Dharmacon Cat. No. 64116 |
| Fluozin-3 AM Thermo Fisher Cat. No. F24195 |
| Fluoxetine hydrochloride Tocris Bioscience Cat. No. 0927/50 |
| PowerUp^TM^ SYBR^TM^ Green Master Mix Thermo Fisher Cat. No. A25742 |
| TaqMan^TM^ RNA-to-CT^TM^ 1-Step Kit Thermo Fisher Cat. No. 4392938 |
| ProLong^TM^ Gold Antifade Mountant Thermo Fisher Cat. No. P36934 |
| Paraformaldehyde Sigma-Aldrich Cat. No. P6148 |
| Sodium chloride Merck Cat. No. DJ9D692758 |
| Potassium dihydrogen phosphate GR Merck Cat. No. ML9M592984 |
| di-Sodium hydrogen phosphate anhydrous Merck Cat. No. QB5Q650227 |
| Potassium chloride Sigma-Aldrich Cat. No. P9541 |
| PVDF Blotting Membrane GE Healthcare Life Sciences Cat. No. 10600023 |
| Methanol Merck Cat. No. SB0SF70138 |
| **Experimental Models: Cell Lines** |
| Human Hepatoma cell lines (Huh-7) Japanese Collection of Research Cat. No.  Bioresources Cell Bank JCRB0403 |
| Human Plateable Hepatocytes Thermo Fisher Cat. No. HMCPTS |
| Baby hamster kidney cells-21 (BHK-21) (C-13) ATCC Cat. No. CCL-10 |
| BHK-21 cells stably expressing DENV-2 replicon  A kind gift from Radhakrishna Padmanabhan (Boonyasuppayakorn et al., 2014) |
| **Oligonucleotides** |
| ZnT1­­_F: TCGAAATGTTGAAGGAGTTG Pre-designed Sigma-Aldrich |
| ZnT1_R: ACATTTTATGTGAGCAGTGG Pre-designed Sigma-Aldrich |
| ZnT2_F: CACTGTGACCCCAAGAAGGG Pre-designed Sigma-Aldrich |
| ZnT2_R: CAGGTACCCACCAACGACTTC Pre-designed Sigma-Aldrich |
| ZnT3_F: TCTATCTCTCCATCCCTCTG Pre-designed Sigma-Aldrich |
| ZnT3_R: TTTTTGCAGTGAGACACAAG Pre-designed Sigma-Aldrich |
| ZnT4_F: AAGCCAGAATACAAGATTGC Pre-designed Sigma-Aldrich |
| ZnT4_R: CTACTGTATCCCATATGATTCG Pre-designed Sigma-Aldrich |
| ZnT5_F: AACACCAGATAATTGGATCAC Pre-designed Sigma-Aldrich |
| ZnT5_R: AAAGGACAGTATTCTGTTTTCC Pre-designed Sigma-Aldrich |
| ZnT6_F: AGTCTTACTCCAGACAACAC Pre-designed Sigma-Aldrich |
| ZnT6_R: CATTTGTTCATTGGCATCTC Pre-designed Sigma-Aldrich |
| ZnT7_F: CAGTCATTCCCTCTTTAATGG Pre-designed Sigma-Aldrich |
| ZnT7_R: TCATGAGAATGAAAGTGTCC Pre-designed Sigma-Aldrich |
| ZnT8_F: CAGAGTATAAAATAGCCGACC Pre-designed Sigma-Aldrich |
| ZnT8_R: ACCTTCCATGAGTAAGATGG Pre-designed Sigma-Aldrich |
| ZnT9_F: GTAATGGAAAGTCGTGATCC Pre-designed Sigma-Aldrich |
| ZnT9_R: TATAGAAGTAAGGCCCATGC Pre-designed Sigma-Aldrich |
| ZnT10_F: GATGAGTAAACTCTCTGCTG Pre-designed Sigma-Aldrich |
| ZnT10_R: CTTAGGATACTTGATGTGCAG Pre-designed Sigma-Aldrich |
| ZIP1_F: TGAGCCTAGTAAGCTGTTTCGC Pre-designed Sigma-Aldrich |
| ZIP1_R: CAGGGCCTCATCTATGGCA Pre-designed Sigma-Aldrich |
| ZIP2_F: CAGAAGTTCATGGTGCAGAACAG Pre-designed Sigma-Aldrich |
| ZIP2_R: CACTGTCGATCCTCCAGCAG Pre-designed Sigma-Aldrich |
| ZIP3_F: CTGTGAGGGAAAAGCTCCAGAA Pre-designed Sigma-Aldrich |
| ZIP3_R: CGTTGAAGGTCTCCAGGTCG Pre-designed Sigma-Aldrich |
| ZIP4_F: CAAGACGGCCTGCGTAGATA Pre-designed Sigma-Aldrich |
| ZIP4_R: GTCCACGAAGTACTGAGGGC Pre-designed Sigma-Aldrich |
| ZIP5_F: GATCTACTATCTGCCCTGC Pre-designed Sigma-Aldrich |
| ZIP5_R: AGATGTAGCAGTGCATCC Pre-designed Sigma-Aldrich |
| ZIP6_F: TTTGCTGTTCTACTAAAGGC Pre-designed Sigma-Aldrich |
| ZIP6_R: AGGTACCATATCAACCAGAG Pre-designed Sigma-Aldrich |
| ZIP7_F: TCTTGAACCTCATTCTCACC Pre-designed Sigma-Aldrich |
| ZIP7_R: ACAAATTTCTCCACGACAAG Pre-designed Sigma-Aldrich |
| ZIP8_F: AATGATTGACAAAGCCCAAC Pre-designed Sigma-Aldrich |
| ZIP8_R: TTCATGGTTTCAAAAGGGTG Pre-designed Sigma-Aldrich |
| ZIP9_F: CAGTTAATTGTGTTTGTGGC Pre-designed Sigma-Aldrich |
| ZIP9_R: TTTACTGCTCTTACTCAAGTCC Pre-designed Sigma-Aldrich |
| ZIP10_F: AGGAGATTTTGCAGTTCTTC Pre-designed Sigma-Aldrich |
| ZIP10_R: CAAAGTGTGATGTTATTGGC Pre-designed Sigma-Aldrich |
| ZIP11_F: AGAATGGTGAGGCATATCAG Pre-designed Sigma-Aldrich |
| ZIP11_R: CTCTGGAACGTTGTGTATAG Pre-designed Sigma-Aldrich |
| ZIP12_F: AGTGTATGGAAACCAAAACG Pre-designed Sigma-Aldrich |
| ZIP12_R: TATTCTGTAAAGTAGTCTGGGG Pre-designed Sigma-Aldrich |
| ZIP13_F: CAAGCTGCAACTCTCAAC Pre-designed Sigma-Aldrich |
| ZIP13_R: GAGAACACCCAACTACTCC Pre-designed Sigma-Aldrich |
| ZIP14_F: CTGATATGATGGAGTTTTGCTC Pre-designed Sigma-Aldrich |
| ZIP14_R: GACAGGATAATCGCTTCAAC Pre-designed Sigma-Aldrich |
| GAPDH_F: CCACTCCTCCACCTTTGAC Pre-designed Sigma-Aldrich |
| GAPDH_R: ACCCTGTTGCTGTAGCCA Pre-designed Sigma-Aldrich |
| **Software and Algorithms** |
| Prism GraphPad (version 7.0e) |
| cellSens Olympus |
